# Optimizing Access to Unrelated Donors in Canada: Re-Examining the Importance of Donor Factors on Outcomes Following Hematopoietic Cell Transplantation

**DOI:** 10.3390/curroncol31050190

**Published:** 2024-04-30

**Authors:** Gaganvir Parmar, Matthew D. Seftel, Kathy Ganz, John Blake, Jelena L. Holovati, David S. Allan

**Affiliations:** 1 Stem Cells, Canadian Blood Services, Ottawa, ON K1Z 7M3, Canadajohn.blake@dal.ca (J.B.); jelena.holovati@ualberta.ca (J.L.H.); 2 Faculty of Medicine, University of Toronto, Toronto, ON M5S 1A8, Canada; 3 Department of Medicine, Faculty of Medicine, University of British Columbia, Vancouver, BC V1Y 1T3, Canada; 4 Department of Industrial Engineering, Dalhousie University, Halifax, NS B3H 4R2, Canada; 5 Department of Laboratory Medicine & Pathology, University of Alberta, Edmonton, AB T6G 2R3, Canada; 6 Department of Medicine and Biochemistry, Microbiology & Immunology, Faculty of Medicine, University of Ottawa, Ottawa, ON K1H 8M5, Canada

**Keywords:** hematopoietic cell transplantation, allogeneic, donor, selection, registry, outcomes

## Abstract

HLA-matched allogeneic hematopoietic cell transplantation (HCT) is a curative therapy for many patients. Unrelated HLA-matched donors are the most frequently used donor for HCT. When more than one donor transplant option is available, transplant centers can select donors based on non-HLA factors. With improved ability to prevent and treat immune complications, such as graft-versus-host disease and infections, it may be possible to proceed more often using HLA-mismatched donors, allowing greater consideration of non-HLA factors, such as donor age, CMV serostatus, and ABO blood group matching, which have demonstrated important impacts on transplant outcomes. Additional factors to consider are donor availability rates and the usage of domestic donors to optimize outcomes. A review of non-HLA factors and considerations on the selection of optimal unrelated donors for HCT are provided within this updated current context.

## 1. Introduction

Hematopoietic cell transplantation (HCT) is an important therapeutic option for a wide range of diseases, including hematologic malignancies, bone marrow failure syndromes, and inherited immune and metabolic disorders [1,2]. Allogeneic HCT is limited by the identification of suitable hematopoietic stem cell donors. Increasing reliance on healthy, unrelated adult volunteer donors has been driven by reduced access to HLA-matched sibling options due to smaller family sizes [3] and increased preference for younger donors [4,5]. With the expansion of the global pool of volunteer stem cell registrants and a greater focus on recruiting registrants from diverse ethnic groups [6], transplant centers may encounter multiple potential donor options for some patients. Optimal donor selection based on factors that can improve transplant outcomes is worth revisiting in light of improved transplant practices, including more effective strategies to prevent graft-versus-host disease (GVHD) using anti-thymocyte globulin (ATG) [7,8,9] or post-transplant cyclophosphamide (PTCy) [10,11]. In this review, we provide a brief comparison of allogeneic HCT activity in the Canadian context, summarize published data concerning donor factors that impact transplant outcomes, and provide an analysis of the Canadian Blood Services Stem Cell Registry (CBS SCR) composition as it relates to updated considerations for donor selection.

## 2. Evolving Trends in Allogeneic Transplantation: The Canadian Context

Data on allogeneic HCT were recently described for the majority of transplants occurring in Canada between 2000–2019 [12]. There are sixteen centers in Canada that perform allogeneic transplants, including four centers focusing only on pediatric transplantation. Data from 15 centers indicate allogeneic HCT in Canada is increasing, with 4164 transplants reported and included in the analysis from 2010–2019, where 17.2% of recipients were pediatric and 57.9% were male [12]. An HLA-matched related donor was used in 36.6%, down from 54.6% in the period from 2000–2009. HLA-matched unrelated donors became the most common donor source, used in 56.3% of the more recent cohort (2010–2019), while related haploidentical donors and cord blood donors were used in 6.1% and 6.5% of cases between 2010–2019. These data correlate well with international trends. The Centre for International Blood and Marrow Transplant Research (CIBMTR) reported on transplantation trends in the US over the same time period [13], confirming that MUDs have been used more commonly than MRDs since 2010 and account for 36–44% of transplants since 2010, while HLA-mismatched donors (7–10% in US compared to 0.5% in Canada) and haploidentical donors (4–23% in US compared to 6.1% in Canada) appear to be used more often in the US compared to Canada over the same time period [12,13]. Similar usage trends were recently published by the European Blood and Marrow Transplant (EBMT) Registry, reporting data from 694 European centers where increasing use of allogeneic HCT was observed and increased use of unrelated donors and haploidentical donors were described, with decreasing usage of cord blood as a cell source [14]. The EBMT report indicated that 58% of allogeneic transplants were for myeloid diseases and 28% for lymphoid diseases, with 13% of allogeneic transplants performed for non-malignant diseases. These data correspond closely with indications for transplantation in Canada between 2010–2019 that revealed 56.8% were for myeloid diseases, 23.9% for lymphoid disease, and 11.8% for non-malignant diseases [12]. In comparing trends in Canadian transplantation during the period 2000–2009 to the period 2010–2019, an increasing number of allogeneic transplants were performed, an increasing proportion of transplants used unrelated donors, and an increasing age of allogeneic transplant recipients was observed in the more recent decade [12]. 

## 3. Selecting the Optimal Donor

In light of recent innovations, such as the use of post-transplant cyclophosphamide (PTCy) [10,11,15] and anti-thymocyte antiglobulin (ATG) [7,8,9] that can improve the outcome of HLA-mismatched and haploidentical transplantation, it is timely to reconsider donor factors that may influence the selection of optimal allogeneic donors, relying on evidence from more recent studies.

### Role of HLA Matching

HLA matching continues to play a central role in the selection of donors for allogeneic HCT. Standard HLA matching involves HLA-A, -B, -C, and -DR loci (8/8 match), with a preference for including -DQ (10/10 match) [16]. In addition, recent studies support the permissiveness of many HLA-DP mismatches [17], and some centers may perform additional extending HLA typing to further evaluate HLA-DP. 

In cases where neither related or unrelated HLA-matched donors are available, transplant centers can consider alternative donor options that include haploidentical donors (where only one of the two haplotypes that contain the HLA genes is identical) from family members (siblings, children, or parents), HLA-mismatched unrelated donors (either 7/8 or 9/10 HLA mismatch, depending on degree of HLA matching desired by the transplant center), or umbilical cord blood (UCB) transplantation [18]. UCB transplants may comprise two units to achieve an adequate total nucleated cell number and total CD34 count [19]. HLA-matched unrelated donors have historically been associated with superior outcomes in studies compared to HLA-mismatched unrelated donors [20], although the use of PTCy in mismatched unrelated donor transplants may yield favorable outcomes [15] and warrant more study in randomized controlled studies. Ethnic disparities in donor registry composition combined with unfavorable haplotype frequencies within certain ethnic groups result in disproportionate access to unrelated donor matching for certain ethnicities [21]. In these situations, an HLA-mismatched unrelated donor, related haploidentical donor, or cord blood transplant may be needed more often to allow patients to proceed with transplant. 

## 4. Donor Factors to Consider beyond HLA

### 4.1. Donor Age 

Younger unrelated donor age is associated with improved overall survival, as noted in a recent large study using the CIBMTR database [5]. Indeed, age was the only donor factor identified in the validation cohort from that study that was associated with improved disease-free survival. Consequently, in recent years, unrelated donor registries have been focusing on recruiting younger registrants to support improved transplant outcomes. During instances where multiple donor options are available for a patient, a younger, available donor, regardless of other non-HLA factors, is preferred. In an era where older patients are increasingly undergoing transplantation, there may be a choice between an older matched sibling and younger unrelated donors. Recent observational data support that the use of younger unrelated donors is associated with reduced rates of relapse and improved disease-free survival in the context of transplantation for AML and ALL [22,23]. Prioritizing the use of younger donors will continue to motivate registries to recruit younger donors. 

### 4.2. Donor Sex at Birth and Parity Status of Female Donors

Previous studies on the impact of donor biological sex at birth on transplant outcomes indicated that multiparous female donors modestly increased the incidence of GVHD in male recipients compared with male donors for male transplant recipients, leading some organizations to recommend the selection of male donors [24] and some registries to adopt strategies targeting male recruitment. The impact on overall survival, however, does not appear significant. Moreover, rates of GVHD in transplants involving female donors with no more than one prior pregnancy were similar to GVHD rates associated with male donors [25]. A more recent large registry-based study did not find an association between donor sex and overall survival at 2 years [5], leaving donor age as the only significant factor associated with overall survival. The yield of total nucleated and CD34+ cells can be higher with male compared to female donors, which may be relevant in cases of significant weight discrepancy between recipient and donor [24]. With increased awareness regarding the lack of significant difference in transplant outcomes between male and female donors, usage of female donors has increased slightly from the CBS SCR in recent years. In total, 76.8% of donors selected from the CBS SCR were male between 1 April 2019 and 1 April 2020, while only 71.4% of donors were male in the period April 2020 to April 2022 [26]. 

### 4.3. Donor CMV Status

Observational data suggest that donor/recipient sero-concordance is associated with improved outcomes after transplant (i.e., +/+ or −/−) [27]. In one large study of patients receiving ATG-based conditioning, however, donor CMV serostatus had no impact on overall survival in patients who were CMV-negative. Moreover, sero-positive patients had worse survival when transplanted with a CMV sero-negative donor, confirming that CMV matching in seropositive patients can improve outcomes [28]. However, baseline CMV serostatus is not available for many registrants unless they have previously undergone this testing as a blood donor or have submitted blood samples in the past for confirmatory HLA typing and had concomitant infectious disease screening tests. Some registries are increasingly providing this information at the time of donor searching based on CMV serological testing from buccal swabs, but the likelihood of negative serostatus switching to seropositivity still needs to be considered. It remains unclear whether a CMV sero-concordant unrelated donor is preferred over a sibling donor with unfavorable CMV mismatching. 

### 4.4. Donor ABO Status

Donor ABO status has become less of a concern in the era of peripheral blood progenitor cell transplantation as the hematocrit of the progenitor cell product is often well below 8%, making clinically relevant hemolysis of incoming donor cells unlikely in the event of a major ABO mismatch. In cases of a major ABO mismatch, measuring anti-A and anti-B titers in the recipient and reducing recipient isohemaglutinins by plasmapheresis is one preventative approach [29]. Some transplant centers perform red cell reduction of the cell product, especially if the cell source is bone marrow [30], which has a greater hematocrit compared with peripheral blood progenitor cell products. A recent study by CIBMTR addressing ABO mismatch on transplant outcomes in patients with acute leukemia receiving related or unrelated donor transplants was recently reported [31]. Major ABO mismatch occurred in approximately 25% of transplants and was associated with worse overall survival (HR 1.16, 1.05–1.29, *p* = 0.005), inferior platelet engraftment, and increased risk of primary graft failure (HR 1.60, 1.12–2.30, *p* = 0.01). Relapse and GVHD were not impacted. Curiously, donor age was not associated with outcomes but was not available for many of the patients included in the study. If these results can be replicated by others, it may lead to renewed interest in donor–recipient ABO matching and could lead some transplant groups to assign higher priority to this variable when selecting among multiple HLA-matched young donor options.

### 4.5. Cytokine Profiles, KIR Matching, and Other Factors

Smaller studies have addressed the role of donor cytokine levels as factors that could influence transplant outcomes in both unrelated and related allogeneic HCT. In one study of 315 HLA-matched transplants, a panel of 22 candidate cytokine genes revealed that the -1082GG variant of the IL10 gene was associated with a three-fold reduction in grade II–IV aGVHD and improved overall survival with no increase in relapse [32]. The variant was associated with increased IL10 production and attenuated CD8+ T cell reconstitution. An examination of chemokine system gene polymorphisms in 1370 HLA-matched donor and recipient pairs revealed that recipient homozygosity for the CCR5 (H1/H1) haplotype was associated with improved disease-free and overall survival compared to other genotypes [33]. Curiously, the same donor CCR5 genotype did not confer the same benefit. Moreover, gene expression profiling of donor CD4 and CD8 T cells prior to transplant in 50 cases identified a “dangerous donor” profile associated with expression levels of specific genes, including Smad3, which influences the regulation of TGF-beta signaling and cell proliferation and was associated with increased incidence of chronic GVHD [34]. A study of killer immunoglobulin-like receptor (KIR) genotypes in 281 HLA-matched transplants undergoing ATG-based conditioning regimens identified that KIR genotype mismatching between donor and recipient was associated with increased cGVHD but not overall survival [35]. The presence of donor clonal hematopoiesis has been recently studied, and in one study, clones of variant allele fraction greater than 1% containing DNMT3A mutations were associated with improved progression-free and overall survival in a cohort of 256 transplant recipients [36]. More studies of various cytokine variants and other important gene mutations appear warranted to consider more precisely the potential role of donor genome profiling as part of donor selection, including both related and unrelated donors. Registries do not typically provide this information when searching for potential donors, and additional tests on the donor would be required. These factors remain under active research, and consideration of these factors in donor selection will require additional study and validation. 

### 4.6. Selection and Use of Domestic Donors

Ideally, transplant centers should have access to suitably HLA-matched, readily available domestic donors for their patients, as opposed to importing donor products from international registries. However, the goal of domestic self-sufficiency remains unrealistic for most jurisdictions, given the ethnic complexity in most populations and subsequent reliance on the global pool of registrants. In addition to the importance of HLA matching, non-HLA-based factors are important factors that influence donor selection by transplant centers, including donor age, CMV serostatus, and ABO group. In an analysis of donor selection patterns by Canadian transplant centers, approximately 25% of transplant recipients with a potential HLA-matched domestic donor will proceed with an international donor, driven largely by non-HLA factors [37]. Over time, however, Canadian transplant recipients are able to access HLA-matched donors at a higher frequency, and, in particular, non-white recipients are increasingly able to access better HLA-matched donors and better-matched cord blood units in 2018 compared to 2013 [38]. At the outset of the recent global pandemic, Canadian transplant centers performed increased numbers of HLA verification typing requests and activated more donor workups on Canadian donors compared to the pre-pandemic era. This suggested greater interest in domestic donor usage to limit logistical risks associated with outbreaks of infections such as COVID-19 [39]. Delays in transplants due to logistical issues caused by the pandemic were associated with adverse outcomes in patients with high-risk disease in a report from a large center in the US [40], further supporting the need for resources and approaches that minimize the risk of delays. Most transplants, however, were able to proceed on schedule despite the pandemic due to swift and widespread adaptations by registries in the US [41], Germany [42], and elsewhere. Potential increased reliance on domestic donors during the pandemic highlights the importance of maintaining and investing in domestic donor registries.

### 4.7. Availability of Donors

Improving the availability of donors has been identified as an opportunity to increase the usage of registrants in many registries. In some cases, contact information has changed, and registrants can no longer be reached. Inability to contact registrants accounts for 43% of unavailability, while personal reasons (28%) and medical ineligibility (19%) were other common causes for being unavailable in a recent analysis of the CBS SCR [37]. Factors associated with greater donor availability were recently published [43], and in multivariable analysis, we identified that recruitment of previous whole blood donors or from blood donation clinics was associated with a 2.5-fold greater availability, perhaps due to more frequent contact and updating of contact information. Online registration was also highly associated with greater availability (3.6-fold greater availability), and consequently, online registration tools have subsequently been enhanced. The CBS registry has moved towards greater online registration, increased recruitment of whole blood donors, and increased frequency of contacting registrants to enhance donor availability at the time of requested donation. 

## 5. Discussion: How Can Registries Reflect the Changing Needs? A Closer Look at the CBS Stem Cell Registry 

In Canada, the Canadian Blood Services Stem Cell Registry (CBS SCR, for Canada, except the province of Quebec) and the Hema-Quebec registry of unrelated donors (for the province of Quebec) facilitate the identification and selection of unrelated donors for patients undergoing allogeneic HCT. The integration of unrelated donor HCT registries worldwide through the World Marrow Donor Association (WMDA), which operates a Search, Match and Connect^TM^ platform for improved donor searching, has facilitated improved donor identification for patients in need (www.wmda.info (accessed on 8 November 2023)). Both the CBS SCR and Cord Blood Bank (CBB) and the unrelated donor registry and cord bank at Hema-Quebec are included within the global inventory managed by the WMDA, which now includes more than 42 million adult registrants and over 800,000 publicly banked umbilical cord blood units [44]. The CBS SCR evolved from initial work by the Bruce Deniston Society in 1988 [45], a group of members of the Royal Canadian Mounted Police force, and grew to a size that contributes significantly to both Canadian and international allogeneic HCT, with a total of more than 440,000 searchable registrants (to 8 November 2023). Of these, 61,730 registrants (~13.9%) have undergone high-resolution typing at 5-HLA loci, representing registrants recruited since 2015. The CBS CBB was launched in 2013, and as of November 2023, a total of 40,000 collections have been completed, with a total of 4351 high-quality units banked, all typed at high resolution at 5 HLA loci; more than 75 units have been distributed for transplantation. 

While the goal of WMDA efforts and registries worldwide is to provide the best possible match for every patient, the likelihood of donor–recipient HLA matching varies across different ethnic groups [21]. For this reason, many registries have embarked on recruitment strategies that optimize ethnic diversity to enhance HLA match likelihoods and allow transplant centers to select optimal donors that consider donor factors beyond HLA matching alone. A current listing of self-reported ethnicities in the SCR and CBB at CBS (to 1 May 2023) is provided in Table 1, and changes over time (Table 2) align with recent recruitment strategies aimed at augmenting the ethnic diversity of donors. The proportion of registrants who self-report as white has decreased since 2018, with concomitant increases in non-white ethnic groups to provide a better reflection of the ethnic composition reported in the 2021 Canadian census [46].

In Canada, there is a growing recognition that a significant proportion of the population identifies as multi-ethnic [47], introducing new challenges to accurately reflect this diversity in HLA modeling work on match likelihoods. To supplement the CBS SCR, the CBS’ CBB was established to improve access for patients of diverse ancestry. Initial analysis of the CBS CBB’s usage demonstrated that 18% of distributed cord blood units were of multi-ethnic background [48]. However, 54% of distributed cord blood units were from donors self-reporting as white, which underscores the ongoing need to collect units from this group to meet patient needs and avoid an overall reduction in total matches.

A degree of “redundancy” in HLA phenotypes in recruited registrants is inevitable but may be variable across ethnic groups. Interestingly, when considering 7/8 HLA mismatches, a high probability of finding suitable donors already exists within current registries across all ethnic groups, as highlighted in a recent analysis by the US-based NMDP registry [10]. Improved outcomes for HLA-mismatched transplantation may be anticipated due to improvements in managing immune-related complications and innovations in the prevention of graft-versus-host disease (GVHD) and infections. [7,8,9,10,11]. Increasing focus on non-HLA factors may arise in the setting of more widespread usage of mismatched donors, although favoring other factors over HLA in donor selection algorithms has not been formally investigated. 

The CBS SCR has recently increased the recruitment of younger donors to align with reports that younger donors improve the survival of HCT recipients [5]. Since 2009, there has been a marked increase in younger donors on the CBS SCR. A total of 224,858 registrants in 2021 were under 36 years old (51% of total registrants), a marked increase from 26% in 2009.

The median age of peripheral blood stem cell donors selected from the CBS SCR between 2019–2022 was 27 years, with the age distribution of these donors provided in Table 3.

While CMV serostatus is only available at the time of HLA verification testing of potential donors from the CBS SCR at this juncture, consideration of strategies to provide this information at the time of searching could prioritize the use of CMV serostatus in selecting donors from the CBS SCR. Moreover, greater recruitment of whole blood donors may provide opportunities to perform CMV testing and make this information available in search reports. Recruitment of whole blood donors may also increase availability as more recent and updated contact information may be obtained.

The CBS SCR and other registries will need to continually adapt to the needs of patients and shifts in donor selection practices based on evidence supporting the impact of donor factors on transplant outcomes. 

## 6. Conclusions and Future Directions: Considerations for Unrelated Donor Selection

While specific recommendations rooted in formal grading of evidence are not available from the limited amounts of recent data in the era of more widespread usage of improved strategies to prevent GVHD, such a systematic approach may be warranted for more precise evaluation of donor factors in the near future. Recent data, however, strongly suggest that HLA-matched donors remain preferable in most cases of allogeneic HCT. Younger donors improve recipient overall survival and should be prioritized. In cases of transplantation for older recipients, the use of younger (<36 years old), unrelated donors compared to older (>50 years) sibling donors appears justified in the context of transplantation for acute leukemia. Donor–recipient CMV matching may be clinically beneficial, but large observational studies did not reveal a consistent impact on overall survival (OS). Donor–recipient ABO mismatch can impact transplant outcomes, although more study is needed given the incomplete data in published retrospective studies. Preferential use of male donors is likely overemphasized, with no difference noted in OS compared to female donors. Based on older data, there is a slightly increased risk of GVHD when using multiparous female donors. The relevance of this during the current era of improved GVHD prevention strategies should be revisited. Other factors such as cytokine profiles, KIR matching, and donor clonal hematopoiesis remain the subject of ongoing research, and incorporation of these factors in donor selection algorithms appears premature. Preferential use of domestic registries may offer economic and practical benefits regarding mitigation of risks when endemic or pandemic infections or other major logistical challenges exist. The analysis of donor factors that impact transplant outcomes requires intermitted revisiting as novel approaches to transplantation emerge.

## Figures and Tables

**Table 1:** Percentage of registrants in Canadian Blood Services’ Stem Cell Registry and Cord Blood Bank by self-reported ethnic groups (to 1 May 2023).

Ethnicity (Self-Reported)	Stem Cell Registry (%)	Cord Blood Bank (%)
White	65.9	36.6
South and Southeast Asian	9.9	22.3
Chinese	7.1	3.6
Multiple ethnicity	4.1	26.0
Black *	1.7	5.3
Arab	1.6	3.6
First Nations, Metis, Inuit	1.5	0.4
Hispanic	1.1	1.2
Other/Unknown	8.6	1.2
TOTAL	100.0	100.0

* includes Black–African, Black–Caribbean, and Black–Other.

**Table 2:** Proportion of major self-reported ethnicity groups in the 2018 and 2023 SCR compared to proportions reported in the 2021 Canadian Census and patient searches from 2021.

Ethnicity (Self-Reported)	2018 SCR Proportion (%)	2023 SCR Proportion (%)	5-Year Delta (% Change)	2021 Census (%)	2021 Patient Searches (%)
White	68.3	65.9	−2.4 (−3.5)	70.0	76.1
Black	1.46	1.66	+0.2 (+13.7)	3.5	3.4
Chinese	7.18	7.14	−0.04 (−0.6)	4.7	6.6
South Asian	6.14	7.04	+0.90 (+14.7)	7.1	10.0
Indigenous	1.31	1.51	+0.20 (+15.2)	5.0	2.8

**Table 3:** Age of donors from the CBS SCR who have donated between 2019–2022.

Donor Age, *n* (%)	Total Donors, *n* = 291
18–25	117 (40.2)
26–35	133 (45.7)
36–45	33 (11.3)
46–55	6 (2.1)
55–65	2 (0.7)
Median	27
Mean ± SEM	28.4 ± 0.4
